# Effectiveness of an individual, online e-learning program about sexually transmitted infections: a prospective cohort study

**DOI:** 10.1186/s12875-017-0625-1

**Published:** 2017-04-24

**Authors:** Linda H. A. Bos-Bonnie, Jan E. A. M. van Bergen, Ellen te Pas, Michael A. Kijser, Nynke van Dijk

**Affiliations:** 1 Department of General Practice, Academic Medical Centre-University of Amsterdam, Meibergdreef 9, Amsterdam, 1105 AZ The Netherlands; 2 Department of General Practice, Academic Medical Centre-University of Amsterdam, STI Aids Netherlands (Soa Aids Nederland), Meibergdreef 9, Amsterdam, 1105 AZ The Netherlands; 3 Department of Educational Support, Academic Medical Centre-University of Amsterdam, Meibergdreef 9, Amsterdam, 1105 AZ The Netherlands; 4 Dutch College of General Practitioners (Nederlands Huisartsen Genootschap, NHG), Mercatorlaan 1200, Utrecht, 3528 BL The Netherlands

**Keywords:** Education, Medical, Continuing [MeSH], E-learning program, Sexually Transmitted diseases [MeSH]

## Abstract

**Background:**

Primary health-care professionals play an important role in the treatment and prevention of Sexually Transmitted Infections (STI). Continuing Medical Education (CME)-courses can influence the knowledge and behavior of health-care professionals concerning STI. We performed a prospective cohort study to evaluate if the individual and online e-learning program “The STI-consultation”, which uses the Commitment-to-Change (CtC)-method, is able to improve the knowledge, attitude and behavior of Dutch General Practitioners (GPs), concerning the STI-consultation. This e-learning program is an individual, accredited, online CME-program, which is freely available for all GPs and GP-trainees in the Netherlands.

**Methods:**

In total 2192 participants completed the questionnaire before completing the e-learning program and 249 participants completed the follow-up questionnaire after completing the e-learning program. The effect of the program on their knowledge, attitude and behavior concerning the STI-consultation was evaluated.

**Results:**

In total 193 participants formulated 601 learning points that matched the learning objectives of the program. The knowledge and attitude of the participants improved, which persisted up to two years after completing the program. Another 179 participants formulated a total of 261 intended changes concerning the sexual history taking, additional investigation and treatment of STI, 97.2% of these changes was partially or fully implemented in daily practice. Also, 114 participants formulated a total of 180 “unintended” changes in daily practice. These changes concerned the attitude of participants towards STI and the working conditions concerning the STI-consultation.

**Conclusion:**

The individual, online e-learning program “The STI-consultation”, which uses the CtC-method, has a small but lasting, positive effect on the knowledge, attitude, and behavior of GPs concerning the STI-consultation.

**Electronic supplementary material:**

The online version of this article (doi:10.1186/s12875-017-0625-1) contains supplementary material, which is available to authorized users.

## Background

Since the incidence of sexually transmitted infections (STI) is rising, the pressure on health-care systems is increasing and the health of many (young) people is at risk [1–4]. Primary health-care professionals therefore, have to be able to provide sufficient care to patients who are at risk for, or suffer from, STI [5]. However, investigation among primary care physicians has shown that few health-care professionals regularly take a sexual history form their patients, and that these histories rarely provide sufficient information for an appropriate diagnostic process, treatment and sexual education and counseling [6].

The knowledge and behavior of general practitioners (GPs) concerning STI can be influenced by continuing medical education (CME)-programs [7]. The goal of CME is to improve the knowledge and competencies of health-care professionals and to change their behavior in daily practice. To achieve these improvements, there has been an increasing interest in the improvement of the quality and effectiveness of CME [8]. Additionally, next to the traditional, offline forms of CME, like lectures and workshops, the use of online CME-courses (e-learning), using the internet and other electronic media to transfer educational programs, is increasing [8–12]. The advantage of e-learning programs for CME over traditional forms of CME is that they are flexible. Courses can be accessed whenever and wherever wanted, and they can be adapted to the entrance-level of the participant [8]. E-learning programs for CME have shown to achieve an enduring increase in knowledge, and induce changes in behavior [9].

To achieve a behavioral change following a CME-course, the Commitment-to-Change (CtC)-method seems a useful addition [13–15]. In the study performed by Domino and colleagues it was suggested that the use of the CtC-method in a CME-course was more likely to induce a behavioural changes than a CME-course not using the CtC-method [13]. Additionally, Pereles and colleagues suggest that the behavioural changes made in reaction to the CtC-method sustained over a longer period of time [15]. The CtC-method stimulates the translation from education to daily practice by focusing on personal plans for behavioral change. One to one-and-a half month after completing the CME-course, participants are reminded of their intended change and asked whether they have changed their practice. It is this change of behavior that is the pursued outcome of the CtC-method [16]. The reminder can be presented to the participants both orally or in writing.

To date, little attention has been devoted to the effect of the CtC-method in e-learning programs. Although e-learning programs for CME are able to improve knowledge and induce changes in behavior [9], there are still are doubts on the role of online CME-courses and the effectiveness of self-directed learning [8]. Additionally, the CtC-method has only been studied in traditional, offline CME-courses [13–15].

When intending to improve the knowledge concerning STI and the sexual history taking-behavior of health-care professionals, a behavioral change has to be achieved. The aim of the present paper is to evaluate if the individual, online e-learning module “The STI-consultation”, using the CtC-method, has a lasting, positive effect on the knowledge, attitude, and behavior of GP’s concerning the STI-consultation.

## Methods

### Context

In the Netherlands, GPs perform 270,000 STI-consultations per year, accounting for 70% of the STI-consultations [17, 18]. CME for Dutch GPs is offered by the Dutch College of GPs (NHG), which develops and manages guidelines and individual CME-programs, and organizes CME-meetings ten times a year [19]. The CME-programs of the NHG are accessible by all GP’s and GP-trainees in the Netherlands. New CME-programs are promoted by e-mail, by mail, and via the website of the NHG.

### Intervention

The individual e-learning program “The STI-consultation”, which uses the CtC-method, was created by the NHG in co-operation with the Dutch expertise-center “STI-aids Netherlands” and the department of General Practice of the Academic Medical Centre-University of Amsterdam. The content e-learning program was based on the content of the NHG-guideline “The STI-consultation” [20]. The e-learning program “The STI-consultation” was constructed using the “Competence profile and attainment targets for GPs” [21–23], which is based on the CanMEDS competency framework [23, 24]. The specific learning objectives for the program per competence area are presented in a separate file [see Additional file 1: Learning objectives for the e-learning program “The STI-consultation”, presented per competence-area.]. The e-learning program is available from the website of the NHG since September 2013. It is freely available to all 12,500 Dutch GPs and GP-trainees, participation in the e-learning program is voluntary. Completing the e-learning program “The STI-consultation” takes about two hours and participants can decide themselves whether they choose to complete the e-learning program in one time or in different attempts. CME-credits will be awarded after the full completion of the e-learning program [25].

Changing the behavior of health-care professionals in daily practice requires three stages of change: confrontation with activities that trigger the consideration of change (priming), experiencing strategies to motivate or facilitate the change (focusing), and maintaining the behavioral change by means of a supportive activity (follow-up) [26–28]. The e-learning program “The STI-consultation” uses various didactic methods to meet the learning objectives of the program and to achieve a behavioral change [21]. The didactic methods of interest in this study were the quickscan (priming), the short and interactive videotaped case-studies with examples of good and less appropriate behavior (priming and focusing) and the CtC-method (follow-up). The questions in the quickscan concerned the knowledge and attitude of participants towards the STI-consultation. The questions used in the quickscan are presented in an additional file [see Additional file 2: English-language version of the questionnaire used in the study]. They were based on the NHG-guideline [29], and on difficulties that GPs experienced during a STI-consultation, obtained by means of a focus group among GPs. The quickscan was both used for triggering the consideration of change (priming) and to evaluate the effect of the e-learning program on the knowledge and attitude of participants about STI. The videotaped case-studies were used to introduce a role model for the GPs. Role models are persons who a learner can identify with, who are in a position that the learner wants to be in, and master skills that the learner wants to master. Role models are able to influence the attitudes and behavior of learners [30–32]. The CtC-method asked participants which change they intended to make as a result of what they had learned in the CME-course. After completing the CME-course, participants were reminded of their intended change two times by e-mail and they were asked if they had implemented the intended change in their daily practice [16]. In order to evaluate the effect of the e-learning program on the knowledge, attitude, and behaviour of participants over time, we compared the results of participants that completed the e-learning program more than one year ago to the results of the participants that completed the e-learning program one year ago or less.

### Study population and data collection

**Fig. 1:**
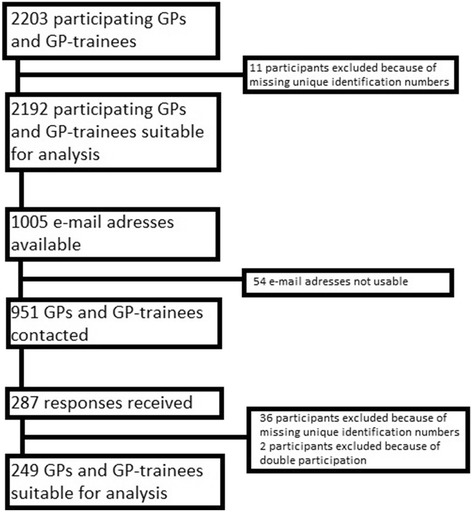
Flowchart of the selection of participants

The e-learning program “The STI-consultation” was freely accessible for all Dutch GPs to allow the full population of GPs to participate in the study. The data for this project were collected among GPs that had completed the program. Data were collected since the e-learning program became available in September 2013 and the data-collection was completed in September 2015. In total 2387 GPs completed the e-learning program, 2203 participants gave permission to collect their data (Fig. 1, Flowchart of the selection of participants). Eleven participants had to be excluded because of missing data, so the data of 2192 participants were used for analysis. Table 1 shows the characteristics of the participants. The mean age of the participants was 38.9 (+/− 9.7) years, 14.8% was GP-trainee. The mean time since the completion of the e-learning module was 18.2 (+/−5.8) months.

**Table 1:** Basic characteristics of participants who completed the e-learning program “The STI-consultation”

Characteristic	Number	Percentage
*Age*		
Mean 38.9+/−9.7 year		
< 30 year	178	10.8%
30–39 year	869	52.3%
40–49 year	313	18.9%
50–59 year	228	13.8%
60–64 year	47	2.8%
> 65 year	19	1.1%
*Gender*		
Male	624	33.6%
Female	1234	66.4%
*Duration of GP-career*		
GP-trainee	161	14.8%
0–5 year	467	42.9%
5–10 year	147	13.5%
10–15 year	110	10.1%
> 15 year	203	18.7%
*Work area*		
Big city	310	28.5%
Medium-big city	331	30.4%
Small city or rural area	447	41.1%
*Duration since e-learning program was completed*		
Mean 18.2+/−5.8 months		
0–6 months	143	8.6%
7–12 months	227	13.7%
13–18 months	362	21.9%
18–24 months	922	55.7%

Participants were asked about their knowledge and attitude towards STI by means of the 11-question quickscan. The answers were given on a 4-point Likert-scale, ranging from “does not apply” (1) to “very applicable” (4). After completing the program, participants were asked to indicate their three most important learning points and their intended change for daily practice both in reaction to what they had learned in the e-learning program.

Two years after the introduction of the e-learning program, in October 2015, participants received an evaluation questionnaire. In total 287 participants completed this questionnaire, 36 participants had to be excluded because of missing data and two participants had to be excluded because of double participation. The responses of 249 (11.4% of original study population) participants were used for analysis. The questionnaire again contained the eleven questions from the quickscan. Participants who formulated intended changes were asked to what extent they had succeeded to implement their intended changes in daily practice. Participants were also asked if they had implemented any “unintended” changes in daily practice. These “unintended” changes were defined as changes that participants made in daily practice in reaction to what they had learned during the e-learning program, without the intention of making the particular change directly after completing the e-learning program. The intended changes and the “unintended” changes were compared to evaluate whether the content of the changes differed.

### Data analysis

To evaluate if completing the e-learning program contributed to a change in the knowledge and attitude of participants towards STI, before —and after-scores on the quickscan were compared. To evaluate if scores changed over time since completing the program we compared groups based on time since completing the program (more than one year ago, or one year ago and less).

The learning points, intended changes and implemented changes in daily practice were analyzed by matching them to the specific learning objectives of the e-learning program [see Additional file 1: Learning objectives for the e-learning program “The STI-consultation”, presented per competence-area.]. If a learning point or change was matched to more than one learning objective, multiple learning points or changes were counted. The matching was done by one researcher (LBB) and checked by another (NvD).

For normally distributed, continuous variables the mean (+/− standard deviation) was calculated. Differences in means between different groups were analyzed by means of ANOVA with the post-hoc Bonferroni-method. Differences in scores over time, were analyzed using the paired sample *T*-test. Nominal values are displayed in cross tables and analyzed using the chi-square test. A *p-*value of <0.05 was considered statistically significant.

Because of the high amount of non-responders in the study we only analyzed the data available to us.

All statistical analyses were performed using SPSS version 22 [33].

## Results

### Knowledge and attitude towards STI

Table 2 shows change on the questions of the quickscan before and after the completing of the e-learning program. Participants showed a statistically significant increase on 5 of the 11 questions. These questions concerned the sexual history taking when patients presented with questions concerning STI, the feeling of competence regarding the STI-consultation, the STI-testing policy in patients under the age of 25 years, the consideration of the diagnosis of HIV in patients with flu-like symptoms, and the persuasion of an active investigation policy in the GP-practice towards HIV-infections.

**Table 2:** Mean Likert-scores on the questions about knowledge and attitude towards STI (*N =* 246)

Moment of answering the questions	Before the e-learning program (Likert (Mean(SD)))	After the e-learning program (Likert (Mean(SD)))	Mean difference (95% confidence interval)	*p-*value
Questions				
I always ask for sexual behavior when a patient presents with questions concerning STI				
	3.5(+/−0.6)	3.6 (+/−0.6)	0.10 (0.01 – 0.19)	0.034^a^
I always ask for sexual behavior when a patient presents with STI-related complaints				
	3.6 (+/−0.5)	3.6 (+/−0.6)	−0.05 (−0.13 – 0.03)	0.202
I find it easy to ask open questions about sexual behaviour				
	3.2(+/−0.6)	3.3 (+/−0.6)	0.05 (−0.04 – 0.14)	0.240
I find it difficult to discuss STI in patients with another cultural background				
	2.3 (+/−0.8)	2.4 (+/−0.7)	0.08 (−0.04 – 0.20)	0.180
When presenting with questions concerning contraception, I always give information about STI and safe sex				
	3.6 (+/−0.6)	3.6 (+/−0.7)	−0.00 (−0.09 – 0.08)	0.919
When presenting for traveler advice, I always give information about STI and safe sex				
	1.8 (+/−0.9)	1.8 (+/−0.9)	0.00 (−0.13 – 0.14)	0.947
I feel competent enough to diagnose and treat STI				
	3.5 (+/−0.5)	3.6 (+/−0.5)	0.10 (0.02 – 0.18)	0.011^a^
I only test for chlamydia in people <25 years of age with no risk factors				
	2.6 (+/−1.1)	3.0 (+/−1.0)	0.37 (0.22 – 0.53)	<0.001^a^
When presenting with flu-like symptoms, I consider the diagnosis of HIV				
	1.6 (+/−0.8)	2.0 (+/−0,9)	0.34 (0.22 – 0.46)	<0.001^a^
I pursue an active investigation policy towards chlamydia-infections in the GP-practice				
	2.4 (+/−0.9)	2.5 (+/−0.8)	0.06 (−0.07 – 0.20)	0.361
I pursue an active investigation policy towards HIV-infections in the GP-practice				
	1.9 (+/−0.9)	2.1 (+/−0.8)	0.22 (0.09 – 0.35)	0.001^a^

^a^Statistically significant differences present

There were no significant differences present between participants who completed the e-learning program less than one year ago and participants who completed the e-learning program more than one year ago (data not shown).

### Formulated learning points after completing the e-learning program

Of the 2203 participants who completed the e-learning program, 193 participants (8.8%) together formulated a total of 601 learning points. Most learning points concerned sexual history taking (28.1%), additional investigation (27.5%), and the treatment of patients with STI (17.5%) (Table 3).

**Table 3:** Amount of formulated *learning points* per competence area and per learning objective

Competence area	Content of learning objectives	Total amount of formulated learning points (N(%))	
*1. Medical Expert Role*		*494 (82.2%)*	
	Sexual history taking		169 (28.1%)
	Epidemiology		49 (8.2%)
	Physical examination		4 (0.7%)
	Additional investigation		165 (27.5%)
	Evaluation		1 (0.2%)
	Treatment		106 (17.6%)
*2. Communicator Role*		*18 (3.0%)*	
	Communication with patient		18 (3.0%)
*3. Collaborator Role*		*8 (1.3%)*	
	Local working arrangements		7 (1.2%)
	Warning of the partner		1 (0.2%)
*4. Manager Role*		*0 (0.0%)*	
*5. Health Advocate Role*		*48 (8.0%)*	
	Sexual education		9 (1.5%)
	Offering investigation for STI		2 (0.3%)
	Cost-conscious working		37 (6.2%)
*6. Scholar Role*		*22 (3.7%)*	
	Test proporties		22 (3.7%)
*7. Professional Role*		*11 (1.8%)*	
	Medical ethics		3 (0.5%)
	Engagement with patient		2 (0.3%)
	Attitude in sexual history taking		6 (1.0%)
*Remaining learning points*		*0 (0.0%)*	
*Total amount of learning points*		*601*	

Didactic methods most frequently used to formulate learning points were the videotaped case-studies and the quickscan. There were no statistically significant differences in knowledge and attitude between participants who did formulate learning points and participants who did not formulate learning points after completing the e-learning program (data not shown).

### Changes in daily practice

In total 179 participants (8.2%) intended to make a total of 261 changes in daily practice (Table 4). Most intended changes concerned the performance of additional investigation (42.5%), the sexual history taking (37.4%), and the treatment of STI (3.8%).

**Table 4:** *Changes* in daily clinical practice per competence area and per learning objective

Competence area	Content of the learning objective	Total amount of formulated intended changes (N(%))		Total amount of implemented “unintended” changes (N(%))	
*1. Medical Expert Role*		*223 (85.4%)*		*142 (78.9%)*	
	Sexual history taking		99 (37.9%)		75 (41.7%)
	Epidemiology		3 (1.1%)		1 (0.6%)
	Physical examination		0 (0.0%)		1 (0.6%)
	Additional investigation		111 (42.5%)		57 (31.7%)
	Evaluation		0 (0.0%)		3 (.7%)
	Treatment		10 (3.8%)		5 (2.8%)
*2. Communicator Role*		*6 (2.3%)*		*3 (1.7%)*	
	Communication with patient		6 (2.3%)		2 (1.1%)
	Counceling		0 (0.0%)		1 (0.6%)
*3. Collaborator Role*		*5 (1.9%)*		*2 (1.1%)*	
	Local working arrangements		0 (0.0%)		1 (0.6%)
	Referral for specialistic treatment		3 (1.1%)		0 (0.0%)
	Warning of the partner		2 (0.8%)		1 (0.6%)
*4. Manager Role*		*7 (2.7%)*		*2 (1.1%)*	
	Testing materials		7 (2.7%)		2 (1.1%)
*5. Health advocate Role*		*10 (3.8%)*		*8 (4.4%)*	
	Sexual education		4 (1.5%)		5 (2.8%)
	Offering investigation for STI		0 (0.0%)		1 (0.6%)
	Awareness of the impact of STI		1 (0.4%)		0 (0.0%)
	Cost-conscious working		5 (1.9%)		2 (1.1%)
*6. Scholar Role*		*3 (1.1%)*		*3 (1.7%)*	
	Test proporties		3 (1.1%)		3 (1.7%)
*7. Professional Role*		*1 (0.4%)*		*3 (1.7%)*	
	Attitude towards sexuality and STI		1 (0.4%)		1 (0.6%)
	Attitude in sexual history taking		0 (0.0%)		2 (1.1%)
*Remaining formulated intended changes*		*6 (2.3%)*		*17 (9.4%)*	
	Change in working conditions STI-consultation		*4 (1.5%)*		*17 (9.4%)*
	Study NHG-guideline “The STI-consultation”		*2 (0.8%)*		*0 (0.0%)*
*Total amount of changes*		*261*		*180*	

*Chi-square test: 15.2, p-value: 0.03*

Participants that formulated intended changes, reported that 34.3% of the intended changes was fully implemented, 62.9% was partially implemented, and 2.8% was not implemented. Reasons for partially or not implementing the intended changes concerned forgetting the intended changes (50.0%), small amount of consultation for STI in daily practice (21.4%), time pressure (14.3%), shame or resistance to talk about STI (7.1%), and the costs of STI-tests (7.1%).

In total 114 participants made a total of 180 “unintended” changes in daily practice. These changes mostly concerned the sexual history taking (41.7%), the additional investigation (31.7%), and a change in the working conditions of the STI-consultation (9.4%). Participants described the change in the working conditions concerning the STI-consultation, as:

*“I do not make a phone-call anymore when a patient has a question about STI, but I want to see the patient in my office, so I can more easily ask the right questions.” (participant)*

*“We extended the duration of a STI-consultation from 20 minutes to 30 minutes. Quality-improvement!” (participant)*

## Discussion

### Summary

This study shows that the individual online e-learning program “The STI-consultation”, which uses the CtC-method, is able to positively influence the knowledge, attitude, and behaviour of GPs concerning the STI-consultation. This positive influence remains up to two years after completing the e-learning program. Participants also noted that the e-learning program had a lasting influence on their knowledge: *“It was an interesting CME-course, and it seems like much information has stayed with me.”(participant).*

Our results indicate that after completing the program, the knowledge and attitude of participants improved and that they changed their behaviour, both by means of intended and “unintended” changes. Remarkable is the finding that, although the amounts are small, the “unintended” changes relatively often concern the working conditions of the STI-consultation and the attitude of participants towards sexuality, STI and sexual history taking, when compared to the intended changes, such as became clear from this remark: *“In sexual history taking, I try to focus more on the sexual behavior of the patient, instead of asking if a patient is homosexual (behavior versus identity)”. (participant).*

### Comparison with existing literature

Our findings suggest that, next to the traditional forms of CME, individual and online forms are effective in improving STI-knowledge and attitudes of health-care professionals. This supports the findings of previous research on the effectivity of internet-based CME and CME for STI [7, 9]. Since health-care professionals, also in our study, still feel resistance to talk about STI or to take a sexual history [34–36], offering an individual e-learning program that participants can complete in their own environment and at their own pace [37], may take away a barrier for completing a CME course on STI, thereby offering the opportunity to improve the knowledge, attitude, and behaviour of health-care professionals concerning the STI-consultation.

The videotaped case-studies are an important feature of the e-learning program. Previous research shows that both the use of the movies or videotaped case-studies and role models are able to contribute to the process of acquiring knowledge and changing attitude [31, 38, 39]. This might especially hold for programs concerning STI, since a videotaped case-study with a role model can show that, when treating a patient in an open, respectful, and unbiased manner, questions concerning the sexual behaviour of the patients are accepted and answered by the patient. The participants also considered the videotaped case-studies to be important for the e-learning program: *“The videotaped case-studies are a nice way to support the sexual history taking of doctors.” (participant).*

In this study we have also seen that participants formulated intended changes for daily practice, of which 97.2% was partly or fully implemented in daily practice. This does not only support the findings of previous research performed on the use of the CtC-method in traditional CME-courses [13–15], but it also indicates that the CtC-method might be effective in individual online CME-courses. Interestingly, participants also made changes without intending to make that particular change in advance. This phenomenon may be explained by individual differences in the priming-stage of the process of making an change in daily practice. In the priming-stage, a certain degree of dissatisfaction with the events in daily practice has to emerge in order to be able to make a change in daily practice. Not every person recognizes or accepts the signals of dissatisfaction at the same time, leading to individual differences in the process of change in daily practice [28]. This is confirmed by the content of these “unintended” changes, which are more frequently related to changes in the working conditions concerning the STI-consultation and the attitude of the participants towards STI and sexual behaviour. The latter may be explained by the theory that a change in professional behaviour or attitude can only take place when one recognizes the impact of the specific behaviour or attitude on others [40], leading to a change whenever a person again is confronted with the particular situation.

### Strengths

Our study is performed in a large study population of over 2000 GPs. Additionally, we were able to analyze the effect of the e-learning program up to two years after the program was completed, showing that changes induced by individual e-learning can have a long-term effect.

### Limitations

Our study is limited by the use of self-reported data of the participants, possibly leading to socially desirable answers. Especially the self-reported data on behavioural changes in daily practice may be subject to self-report bias, leading to the under-report of behaviour that may be considered as non-desirable by the participants and over-report of behaviour that may be considered as desirable by participants [41]. Additionally, the response-rate for the evaluation questionnaire was low, leading to a considerable amount of missing data and a possible overestimation of the study-results. This low response-rate may be the effect of a few of the disadvantages of e-learning programs, like the lack of social interaction between participants and confusion or frustration as a result of the use of a new and unknown technique [37]. However, analysis of the basic characteristics shows that there are no statistically significant differences between the participants who did respond to the evaluation questionnaire and participants who did not respond to the evaluation questionnaire. It seems that the participants who did respond to the evaluation questionnaire are a good representation of the original study population. Another limitation in our study is the small effect-size in the results of the quickscan. This may be caused by the fact that only a 4-point Likert-scale was used, and by the fact that some of the scores on the questionnaire were already very high before the start of the program. Because we did not use a control group, it is not possible to determine whether the effects we found can be fully attributed to the e-learning program. We are not informed on additional CME-courses that participants followed and the effect of experiences in daily practice on knowledge, attitude and behaviour are difficult to determine.

### Implications for education and research

Our results provide insight in the effectiveness of individual, online CME-courses. Since online CME-courses are gaining popularity [8, 9], it is important to gain more knowledge on their efficacy. Future research could benefit from an intensive comparison of online, individual CME-courses and traditional CME-courses, especially on the STI-consultation, to further determine the role that could be awarded to individual, online CME-courses. Additionally, we could benefit from the use of a control group, non-self-reported data, more detailed response-options for the participants, and a more intensive follow-up procedure to gain more insight in the effects of (components of) individual, online CME-courses on the knowledge, competences, and the behaviour in daily practice of health-care professionals.

## Conclusion

Individual e-learning programs, especially with the addition of CTC, are an effective method for CME, not only for improving and maintaining the knowledge and attitude of health-care professionals, but also for improving their behaviour in daily practice. By offering an individual e-learning program that participants can complete in their own environment and at their own pace, a barrier for completing a CME course on STI may be taken away, also offering the opportunity to improve the knowledge and attitude of the health-care professional concerning STI and sexual history taking.

## Additional files

Additional file 1: Learning objectives for the e-learning program “The STI-consultation”, presented per competence-area (translated from Dutch). This file contains the learning objectives for the e-learning program “The STI-consultation”, presented per competence-area. The content of this file was based on the “Program plan e-learning “The STI-consultation” (Programmaplan PIN “Het soa-consult”). (DOCX 23 kb)

Additional file 2: English-language version of the questionnaire used in the study (translated from Dutch). This file contains the English-language version of the questionnaire used in the study. The content of this file was translated from the original questionnaire used in our study. (DOC 27 kb)

## Abbreviations

CME: Continuing Medical Education
CtC: Commitment-to-Change
GP: General Practitioner
NHG: Dutch College of General Practitioners (Nederlands Huisartsen Genootschap)
STI: Sexually Transmitted Infections
