# Supplementary material for: Effectiveness of an individual, online e-learning program about sexually transmitted infections: a prospective cohort study
Source: BMC Fam Pract. 2017 Apr 24;18:57. doi: 10.1186/s12875-017-0625-1 (PMC5402660; doi:10.1186/s12875-017-0625-1)
Supplement: Supplementary file 1 — Learning objectives for the e-learning program “The STI-consultation”, presented per competence-area (translated from Dutch). This file contains the learning objectives for the e-learning program “The STI-consultation”, presented per competence-area. The content of this file was based on the “Program plan e-learning “The STI-consultation” (Programmaplan PIN “Het soa-consult”). (DOCX 23 kb) [file 12875_2017_625_MOESM1_ESM.docx]

**Additional file 1**

Learning objectives for the e-learning program “The STI-consultation”, presented per competence-area (translated from Dutch). Taken over from the “Program plan e-learning “The STI-consultation” (Programmaplan PIN “Het soa-consult”).

| **Learning objective** | **Specific subject** | **Support** | **Didactic method** |
| --- | --- | --- | --- |
| **1. Medical Expert Role** | | | |
| The GP knows the anamnestic questions that need to be asked in order to be able to make a risk calculation for the patient that visits the GP with symptoms of a (possible) STI or questions or fears about STI. | Sexual history-taking | Core message of the NHG-guideline | Casuistry  Videotaped case-study |
| The GP knows that asking for sexual behaviour is essential for the collection of material for additional research, both for the collecting location and the collecting material. | Sexual history-taking | Core message of the NHG-guideline  Focus group research | Casuistry  Videotaped case-study  Knowledge test |
| In the case of a high risk for STI, the GP is alert for other complaints that might be caused by STI. | Sexual history-taking in patients from a risk group population | Core-message of the NHG-guideline | Casuistry  Videotaped case-study  Quickscan |
| The GP knows the epidemiology of STI in his work area. | Epidemiology | NHG-guideline | Quickscan |
| The GP knows what physical examination is essential to perform in a patient with STI-related symptoms. | Physical examination | NHG-guideline | Casuistry  Quickscan |
| The GP knows what specific STI-related investigation has to be considered and/or offered, both in patients with and without STI-related symptoms. | Additional investigation | Core-message of the NHG-guideline | Casuistry  Videotaped case-study  Quickscan |
| The GP knows the variety of STI-tests, materials of STI-tests and the different test-sites and collection-techniques. | Additional research, test-equipment | Core-message of the NHG-guideline  Focus group research | Casuistry  Videotaped case-study  Knowledge test |
| The GP is able to instruct a female patient to independently collect a vaginal sample. | Instruction test-technique | Focus group research | Thuisarts.nl (website) |
| The GP is able to calculate the risk for STI based on the story of the patients and the findings in physical examination. | Evaluation | Core-message of the NHG-guideline | Casuistry |
| The GP is able to make a diagnosis on the cause of the STI and/or the clinical pattern, based on the information he has obtained from the history-taking, risk-calculation, findings in physical examination and findings in STI-related additional investigation. | Evaluation | Core-message of the NHG-guideline | Casuistry  Quickscan |
| The GP is able to instruct the patient on safe sex, STI-related symptoms, complications of STI, and the risk of infecting other persons. | Treatment, awareness | Core-message of the NHG-guideline  NHG-guideline | Casuistry  Videotaped case-study |
| The GP knows the medical treatment of an STI (per cause or clinical pattern of STI) | Treatment, medication | Core-message of the NHG-guideline  Table | Casuistry |
| The GP knows how the effect of the treatment has to be evaluated. | Treatment, monitoring | NHG-guideline | Casuistry |
| The GP knows when to perform additional STI-investigation when having a positive STI-test. | Treatment, additional investigation | NHG-guideline | Casuistry  Knowledge test  Quickscan |
| The GP knows the specific policy concerning the partner of a patient with a positive chlamydia-, gonorrhea- or trichomonas-test. | Treatment of the partner of the patient | Core-message of the NHG-guideline | Casuistry  Quickscan |
| The GP takes into account the risk of multi-resistant gonorrhea, when treating a gonorrhea-infection. | Treatment, antibiotic resistence | NHG-guideline | Casuistry |
| The GP knows which patients he can and cannot treat himself (HIV, syphilis, pregnant woman) | Treatment of a specific patient group | NHG-guideline | Casuistry  Quickscan |
| **2. Communicator Role** | | | |
| The GP is able to clearly inform the patient about the cause, the course and the treatment of STI, adapted to age, gender, ethnic-cultural background and emotions of the patient. | Communication with the patient, techniques | NHG-guideline | Casuistry  Videotaped case-study |
| The GP is able to explain and gain insight in the risk factors for STI. | Communication with the patient, risk-communication | NHG-guideline | Casuistry  Videotaped case-study |
| The GP is able to help the patient (without complaints) making a well-motivated choice for STI-investigation. | Counceling | NHG-guideline | Casuistry  Videotaped case-study |
| The GP takes into account the specific ethnic-cultural-related aspects of STI (offering STI-testing to patients from HIV-endemic areas, taboos) | Reference-area of the patient | Core-message of the NHG-guideline | Casuistry  Videotaped case-study |
| **3. Collaborator Role** | | | |
| The GP knows the local working arrangements and referral possibilities to other institutions who diagnose (and treat) STI in his work region. | Local working arrangements | NHG-guideline | Checklist |
| The GP knows to what institution a patient with STI can be referred when a referral-indication is present. | Referral for specialistic treatment | NHG-guideline | Casuistry |
| The GP knows the local working arrangements concerning the warning of partner of patients with STI. | Warning of the partner | Core-message of the NHG-guideline | Casuistry  Checklist |
| **4. Manager Role** | | | |
| The GP has access to educational and instructional materials about STI and STI-tests to provide to specific patient groups. | Information material | NHG-guideline | Stocktaking of instructional and testing material in practice |
| The GP has access to different STI-testing materials. | Testing materials | FG  NHG-guideline | Stocktaking of instructional and testing materials in practice  Videotaped case-study |
| The GP is aware of the fact that there is a duty to report a recently acquired hepatitis B-infection. Other STI do not have to be reported. | Mandatory declaration | - | Referral to the National Institute of Public Health and Environment (RIVM) |
| **5. Health advocate Role** | | | |
| Whenever there is a reason (for example: traveler advise or questions about contraception), the GP informs the patient about safe sex and the risk of STI. | Sexual education | NHG-guideline | Questions |
| Whenever there is an indication to talk about STI, the GP easily offers STI-investigation. | Offering investigation for STI | Core-message of the NHG-guideline | Questions  Casuistry |
| The GP is aware of the impact of STI on the person and the overall society. | Awareness of the impact of STI | NHG-guideline | Questions  Casuistry  Infographics |
| The GP avoids applying for non-indicated STI-investigation. Also, the GP knows is able to inform the patient about the price of the different STI-investigations and the reimbursement status in different insurance companies. | Cost-conscious working | NHG-guideline | Questions  Casuistry  Infographics |
| **6. Scholar Role** | | | |
| The GP is aware of the test-properties of the different STI-tests and is able to value the test-outcomes. | Test properties | NHG-guideline | Knowledge test  NHG-guideline “The STI-consultation” |
| **7. Professional Role** | | | |
| The GP respects the differences in sexual norms and values, and takes into account the professional involvement and detachment when taking a sexual history. | Medical ethics | NHG-guideline | Casuistry  Questions |
| The GP is aware of the impact of the diagnosis of STI. | Engagement with patient | NHG-guideline | Casuistry |
| The GP knows his own attitude towards sexuality and his own attitude and motivation towards patients with sexual risk behaviour and inadequate motivation or abilities to change this behaviour. | Attitude towards sexuality and STI | NHG-guideline | Questions  Dilemmas |
| The GP takes into account the feelings of embarrassment and inconvenience in the patient and in himself when taking a sexual history, performing physical examination, and performing specific STI-investigation. | Attitude in sexual history taking | Focus group research | Casuistry |
