# Supplementary material for: Effectiveness of an individual, online e-learning program about sexually transmitted infections: a prospective cohort study
Source: BMC Fam Pract. 2017 Apr 24;18:57. doi: 10.1186/s12875-017-0625-1 (PMC5402660; doi:10.1186/s12875-017-0625-1)
Supplement: Supplementary file 2 — English-language version of the questionnaire used in the study (translated from Dutch). This file contains the English-language version of the questionnaire used in the study. The content of this file was translated from the original questionnaire used in our study. (DOC 27 kb) [file 12875_2017_625_MOESM2_ESM.doc]

**Additional file 2**

English-language version of the questionnaire used in the study (translated from Dutch).

1. I always ask for sexual behavior when a patient presents with questions concerning STI.
   1. Very applicable
   2. Somewhat applicable
   3. Not very applicable
   4. Not at all applicable
2. I always ask for sexual behavior when a patient presents with STI-related complaints.
   1. Very applicable
   2. Somewhat applicable
   3. Not very applicable
   4. Not at all applicable
3. I find it easy to ask open questions about sexual behavior.
   1. Very applicable
   2. Somewhat applicable
   3. Not very applicable
   4. Not at all applicable
4. I find it difficult to discuss (the risk for) STI in patients from a different cultural background.
   1. Very applicable
   2. Somewhat applicable
   3. Not very applicable
   4. Not at all applicable
5. When a patient presents with questions concerning contraception, I always provide information about STI and safe sex.
   1. Very applicable
   2. Somewhat applicable
   3. Not very applicable
   4. Not at all applicable
6. When a patient presents for traveler advice, I always provide information about STI and safe sex.
   1. Very applicable
   2. Somewhat applicable
   3. Not very applicable
   4. Not at all applicable
7. I feel competent enough to diagnose and treat STI.
   1. Very applicable
   2. Somewhat applicable
   3. Not very applicable
   4. Not at all applicable
8. When a patient under the age of 25 years, with no STI-related risk factors and without any STI-related complaints, presents for STI investigation, I advise only to test for chlamydia.
   1. Very applicable
   2. Somewhat applicable
   3. Not very applicable
   4. Not at all applicable
9. When a patient presents with nonspecific, flu-like symptoms, I never really consider the diagnosis of HIV.
   1. Very applicable
   2. Somewhat applicable
   3. Not very applicable
   4. Not at all applicable
10. I consider the contribution of chlamydia to the total burden of disease of the Dutch population big enough to pursue an active investigation policy towards chlamydia-infections in GP-practice.
    1. Very applicable
    2. Somewhat applicable
    3. Not very applicable
    4. Not at all applicable
11. I consider the contribution of HIV to the total burden of disease of the Dutch population big enough to pursue an active investigation policy towards HIV-infections in GP-practice.
    1. Very applicable
    2. Somewhat applicable
    3. Not very applicable
    4. Not at all applicable
